# Recorded spontaneous seizure prior to electroconvulsive therapy after etomidate induction in a patient treated with bupropion: a case report

**DOI:** 10.1007/s40211-025-00553-3

**Published:** 2025-10-13

**Authors:** Evelyn Romina Pircher Nöckler, Laurin Mauracher, Lukas Gasteiger, Imrich Blasko

**Affiliations:** 1 Department of Psychiatry, Psychotherapy, Psychosomatics and Psychotherapeutic Medicine, University Hospital for Psychiatry I, Medical University of Innsbruck, Innsbruck, Austria; 2 Department of Anesthesiology and Intensive Care Medicine, Medical University of Innsbruck, Innsbruck, Austria

**Keywords:** Electroconvulsive therapy, Anesthesia, Etomidate, Bupropion, Spontaneous seizure, Elektrokrampftherapie, Anästhesie, Etomidat, Bupropion, Spontaner Anfall

## Abstract

**Background:**

Electroconvulsive therapy (ECT) is a widely used and effective treatment for refractory depression. Patients hospitalized for ECT regularly take antidepressants or adjuvant antipsychotics, both can influence the quality of seizures. Adaptation of the seizure threshold during an ECT series necessitates adjustments in treatment parameters to ensure adequate seizure quality.

**Methods:**

We report the case of a 46-year-old man with treatment-resistant depression treated with bupropion and ECT, who developed a spontaneous seizure after first-time etomidate anesthesia induction, as previously induced seizures under thiopental were considered insufficient. Serendipitously, an electroencephalogram (EEG) of the seizure was recorded.

**Results:**

The characteristics of the EEG captured after this spontaneous seizure were similar to those of regular ECT seizures under thiopental anesthesia in this patient. After returning to thiopental anesthesia, the remaining ECT course was unremarkable, and the patient’s depressive symptoms improved partially.

**Conclusion:**

Accordingly, the use of etomidate as an anesthesia induction agent for ECT in patients who are concomitantly using bupropion needs to be cautiously considered. The case of a documented etomidate-induced seizure could indicate the relatively benign course of such events.

## Abstract (de)

**Hintergrund:**

Die Elektrokonvulsionstherapie (EKT) ist eine weit verbreitete, wirksame Methode in der Behandlung der therapieresistenten Depression. Begleitmedikation mit Antidepressiva und Antipsychotika während einer EKT-Serie können die Krampfqualität beeinflussen. Krampfschwellen-Veränderung im Verlauf erfordern oft zusätzliche Adaptierungen, um eine suffiziente Anfallsqualität zu gewährleisten.

**Methoden:**

Wir berichten über einen 46-jährigen Mann mit therapieresistenter Depression, der mit Bupropion und EKT behandelt wurde und erstmalig nach Anästhesieeinleitung mit Etomidat einen spontanen Anfall entwickelte, nachdem er aufgrund unzureichend guter Krampfqualität von Thiopental auf Etomidat umgestellt wurde. Ein Elektroenzephalogramm (EEG) des Anfalls wurde aufgezeichnet.

**Ergebnisse:**

Die EEG-Charakteristika während des spontanen Anfalles nach Anästhesieeinleitung mit Etomidat ähneln formal denen der regulären EKT-Anfälle dieses Patienten unter Thiopental-Anästhesie. Nach der Umstellung zur ursprünglichen Anästhesie mit Thiopental verlief die weitere EKT-Serie unauffällig. Es kam zu einer Teil-Remission der depressiven Symptomatik.

**Schlussfolgerung:**

Die Anwendung von Etomidat als Narkoseeinleitungsmittel für die EKT bei Patient:innen, die gleichzeitig Bupropion einnehmen sollte mit Vorsicht abgewogen werden. Dieser dokumentierte Fall eines durch Etomidat ausgelösten Krampfanfalles, könnte auf einen relativ harmlosen Verlauf solcher Ereignisse hinweisen.

## Introduction

Different hypnotics are used to induce general anesthesia during electroconvulsive therapy (ECT), depending on the individual patient’s characteristics. *Etomidate*, an imidazole-based γ(gamma)‑aminobutyric acid type A (GABA_A_) receptor agonist used to induce general anesthesia, differs from other anesthetics by its lack of cardiovascular or respiratory depression. Frequently observed side effects are myoclonic or other involuntary muscle movements, with an unclear link to a possible development of spontaneous generalized seizures [1].

Individual comedication and comorbidities can influence the quality of the ECT seizures and accordingly increase the risk of seizures when etomidate is used simultaneously. Spontaneous epileptic seizures have been documented previously in patients undergoing etomidate anesthesia even without [2, 3] or with concurrent psychiatric medication [4], as have spontaneously developed seizures before ECT application, pointing to patient-intrinsic conditions or comedication [5]. Over the course of an ECT series, the seizure threshold tends to increase, which may lead to inadequate seizures. As using a higher stimulus dose may cause side effects, one alternative for improvement is to change the induction anesthetics. Etomidate, compared to propofol or thiopental, extends the duration of the ECT seizures, with a recent study finding different seizure duration depending on the pharmacotherapy patients received during ECT [6].

In certain populations with an elevated seizure risk (e.g. history of traumatic brain injury, family history of epilepsy, substance use disorder), the frequently prescribed antidepressant bupropion can also trigger seizures [7]. *Bupropion*, a noradrenaline–dopamine reuptake inhibitor, may lower the seizure threshold when co-administered during an ECT course, but may also shorten the seizure duration. It is unclear whether bupropion and etomidate, when used in combination, could have an additive seizure risk or possible synergistic effects. In this context, Dersch et al. reported on a partial status epilepticus after electroconvulsive therapy in a patient on bupropion when anesthesia was performed with etomidate [8].

## Case report

A 46-years-old male with recurrent depressive disorder was admitted after two previous inpatient stays. After clinical stability for several years under escitalopram, the patient relapsed, and the dosage was unsuccessfully increased. Outpatient attempts, including advanced antidepressant therapy trials (escitalopram, sertraline, venlafaxine, mirtazapine, agomelatine, vortioxetine, bupropion) and combination with adjuvant antipsychotics (aripiprazole, quetiapine, olanzapine) under adequate treatment duration and drug monitoring, failed.

On admission, the patient’s current medication was bupropion extended-release (XR) and olanzapine; later, trazodone was added. Nonetheless, the patient reported severe depressive symptoms, with a Montgomery–Åsberg Depression Rating Scale (MADRS) score of 39 points. Next, intranasal application of esketamine was initiated, stopped due to dissociative side effects and poor antidepressant effect, and ECT was considered.

Preliminary examinations prior to ECT, including routinely performed brain magnetic resonance imaging, electroencephalogram (EEG), electrocardiogram (ECG), and blood laboratory studies were unremarkable, with no reported history of traumatic brain injuries or seizures. Before starting the ECT course, olanzapine was tapered and discontinued. Concomitant medications included 150 mg bupropion XR with plasma levels within the therapeutic range and 150 mg trazodone. A series of ECT treatments was started using a Thymatron System IV device (Somatics LLC; Lake Bluff, IL, USA) with 45% of the nominal charge, right unilateral electrode position, the cuff method to record electromyography (EMG), and anesthesia using 400 mg thiopental (5.6 mg/kg) and 70 mg suxamethonium (1 mg/kg). The optimal anesthesia depth before ECT stimulation was assessed with Narcotrend (MT MonitorTechnik GmbH & Co. KG., Hannover, Germany), an EEG-based monitoring device [9]. After initially sufficient seizures under these settings, subsequent stimulations quality criteria decreased, leading consequently to a stepwise augmentation of charge, switch to bitemporal electrode application from the eighth treatment onwards, and extended administration of 0.5 mg flumazenil to lower the seizure threshold. Since at the ninth ECT, no sufficient seizure could be achieved, it was decided to switch from thiopental to etomidate.

At the tenth treatment, 20 mg etomidate (0.3 mg/kg) was applied without using flumazenil, and an ECT in bitemporal electrode application with 150% of the nominal charge was planned. Clinical signs with preserved spontaneous respiration showed inadequate anesthesia depth after induction with etomidate, therefore the muscle relaxant was not administered. Contrastingly, Narcotrend monitored a value under E30, corresponding to a deep general anesthesia [9]. A few minutes post-etomidate, a spontaneous generalized tonic-clonic seizure occurred, with twisting of the bulbs, flexion of the upper extremities, salivation, and masseter spasm. During this spontaneous seizure, anesthesia was deepened with etomidate 10 mg and suxamethonium 80 mg due to absent protective reflexes and inadequate ventilation. Following propofol 40 mg, convulsive activity ceased, and lorazepam was no longer required. No ECT stimulus was applied in this session.

**Fig. 1:**
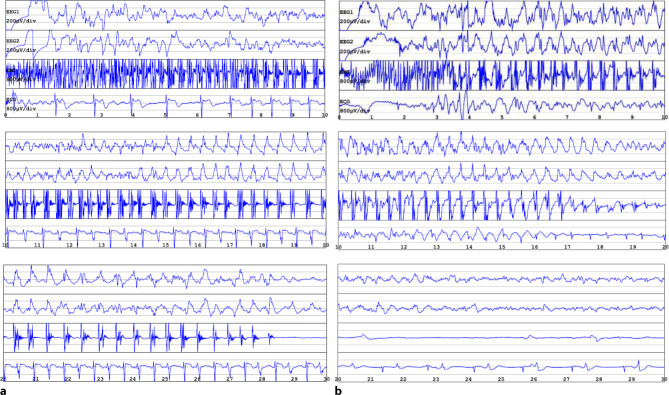
Electroconvulsive therapy (ECT) reports from two different treatment sessions. **a** Spontaneous generalized tonic-clonic seizure after administration of etomidate, 20 mg: intermittent spike activity on the background of low beta and theta activity turning into spike epileptic activity of low amplitude, slow frequency, and transition to medium amplitude (15 to 20 s), medium frequency, with clear seizure termination (second 29) and poor suppression. **b** Induced seizure with 140% energy setting and anesthetic thiopental, 400 mg: intermittent spike activity turns into spike epileptic activity of medium amplitude, medium frequency, with less clear seizure termination and poor suppression. *EEG* *1 and 2* electroencephalogram in right and left channel, respectively. *x‑axis* time in seconds, *y‑axis* amplitude in µV. *EMG* electromyogram. *ECG* electrocardiogram

During the spontaneous seizure, recordings of the EEG, ECG, and EMG were collected (Fig. 1a) and exported to GPD (Genie Patient Database; Elektrika Inc.), a software for digital export of ECT data. The patient recovered without complications, without any subjective symptoms or clinical signs suggestive of neurological dysfunction. A follow-up EEG a few hours later revealed a moderate diffuse cerebral dysfunction consistent with the previous epileptic seizure, with occasional diffuse alternating side-emphasized delta/theta activity and intermittently slowed basal rhythm.

The subsequent two ECT treatments, switched back to thiopental 400 mg, showed adequate ECT-induced seizures (Fig. 1b). After 12 treatments, the ECT course was discontinued due to insufficient response, documented by a MADRS score of 21 points, indicating clinical improvement but an inadequate response to ECT. Bupropion XR was increased to 300 mg.

## Discussion

To our knowledge, this is the first case of a recorded spontaneous seizure after etomidate administration prior to ECT. Patients with a history of epilepsy and/or cerebral cortical lesions appear to be at risk of developing seizures after etomidate administration, while our patient had no identifiable risk factors or known family history of neurological disorders. The EEG after the spontaneous seizure revealed moderate diffuse cerebral dysfunction, without typical signs of epileptic activity. Interestingly, the neuronal activity (Fig. 1a) in the spontaneous seizure under etomidate seems to be comparable to the ECT-induced seizure under thiopental anesthesia (Fig. 1b). The peripheral seizure activity lasted longer, and synchronization and seizure termination seemed to be better in the spontaneous event than in ECT stimulation. Seizure suppression and termination were similar in both convulsions. The dose of etomidate used in our case is comparable to the other cases; administering an additional dose did not result in seizure cessation [4, 5, 10].

The unremarkable further clinical course of our patient after a spontaneous epileptic seizure and continuation of ECT also invite discussion on whether a spontaneous seizure in this context is necessarily cause for concern. Supporting the observation of others, etomidate-induced seizures are rare events; therefore, no change to the ECT procedure may be necessary [4]. In high-risk populations, the use of propofol as anesthetic agent and reduction or discontinuation of certain psychotropic drugs may prevent the occurrence of unpredicted seizures, and routine EEG registration before administering etomidate may be considered [5]. The majority of psychotropic medications can be administered safely along with ECT. However, a continued evaluation of risks versus benefits is indicated, as these substances affect seizure thresholds, seizure propagation, and ECT safety and efficacy. In our case, although the combination of bupropion and etomidate may have contributed to seizure occurrence, no negative impact on the clinical course and treatment of the depressive episode was seen.
